# Acute Ischemic Stroke Following a Camel Bite to the Neck: A Case Report

**DOI:** 10.7759/cureus.95983

**Published:** 2025-11-03

**Authors:** Ahmed A Humaida, Leina Abdalla, Khalid Idriss, Salma B Eltayeb, Osman Ahmedfiqi

**Affiliations:** 1 Internal Medicine, Prince Mohammed Bin Abdulaziz Hospital, Ministry of National Guard Health Affairs, Madinah Munawara, SAU; 2 Internal Medicine, Prince Sultan Armed Forces Hospital, Madinah Munawara, SAU; 3 Intensive Care, Prince Mohammed Bin Abdulaziz Hospital, Ministry of National Guard Health Affairs, Madinah Munawara, SAU; 4 Radiology, Prince Mohammed Bin Abdulaziz Hospital, Ministry of National Guard Health Affairs, Madinah Munawara, SAU

**Keywords:** camel bite, common carotid artery occlusion, stroke, therapeutic anticoagulation, thrombosis of the internal jugular vein

## Abstract

Animal bites are considered one of the leading causes of injuries and even mortality worldwide. While the camel's specific bite power and teeth must be considered when treating these wounds, several clinical characteristics of camel bites are similar to those of more typical animal bite injuries. Ischemic stroke caused by vascular injury is a rare occurrence. Precautions and protective masks for the camel's mouth in late winter and early summer, along with appropriate health education for those handling and caring for camels, can help prevent these injuries. We are reporting a case of a 54-year-old man from the Madinah Munawara area of Saudi Arabia, who had a medical history of diabetes, hypertension, and dyslipidemia, who presented with acute right-sided hemiparesis and dysarthria following a camel bite to the left side of his neck. Imaging confirmed a blockage of the left common carotid artery and thrombosis of the left internal jugular vein. He gradually recovered neurologically after receiving broad-spectrum antibiotics and anticoagulant therapy. This case shows how camel bite injuries can result in severe neurological and vascular complications, including stroke. For better outcomes, immediate imaging and accurate multidisciplinary care are crucial.

## Introduction

The interaction between humans and camels has evolved over the years. The Arabian Peninsula is home to many camels. It became an integral part of these communities' daily existence as a source of food, milk, leather, transportation, sports, and cultural history [1]. It is a peaceful animal that typically obeys its owner. Nevertheless, it has been observed that during the reproductive season, camels become more aggressive and less tolerant of human interaction, increasing the risk of attacks. Those around them are particularly susceptible to their attacks during the breeding season [2]. Injuries to vascular structures of the neck resulting in hemiparesis after camel bites are rarely reported.

We report a case of a 54-year-old man who experienced an acute stroke after being bitten by a camel on his left neck. We highlighted the value of early diagnosis, a multidisciplinary approach, and teamwork to improve patient outcomes in such cases.

## Case presentation

After being attacked by a camel on the left side of his neck, a 54-year-old man with hypertension, diabetes mellitus, and dyslipidemia arrived at our emergency department. He had a transverse submandibular wound on the left side, measuring approximately 7-10 cm in length, with lacerations and puncture marks. The wound was bleeding actively on his arrival. However, he was fully conscious, alert, oriented, and communicative.

On examination, his pulse was 84/minute, blood pressure was 128/82, and SpO₂ was 96% on RA. The Glasgow Coma Scale (GCS) was 15/15, with no neurological deficit (National Institutes of Health Stroke Score (NIHSS) = 0). The wound was cleaned with normal saline irrigation, trimmed, and primarily closed, with a drain placed at the wound's depth. After collecting a wound culture, metronidazole (500 mg every eight hours) and cefazolin (1 g every eight hours) were administered intravenously as an empirical treatment. In addition to wound care and suturing, the patient received tetanus prophylaxis and the first dose of the rabies vaccine as a preventative measure.

The patient developed right-angle mouth deviation, slurred speech, and weakness of the right upper arm two days following the camel bite. His neurological condition worsened, and the patient became confused and agitated. Consequently, he was sedated to control his behavior. The sudden onset of neurological symptoms required further investigations and teamwork with other specialties. The patient was admitted to the intensive care unit (ICU), and urgent imaging with a Neurology consultation for re-evaluation was requested. Neurological evaluation revealed that the patient was alert and oriented, with a mild decrease in level of consciousness (GCS 14/15), and had right-sided hemiparesis, dysarthria, and right facial droop. Blood laboratory tests showed hemoglobin 14.7 g/dL, white blood count 4.49×10⁹/L, platelets 250×10⁹/L, CRP 10 mg/dL, ESR 10 mm/hr, and random blood sugar 8 mmol/L. Additionally, the patient's urea and electrolyte levels, liver function tests, and coagulation screen were all within normal limits.

**Figure 1:**
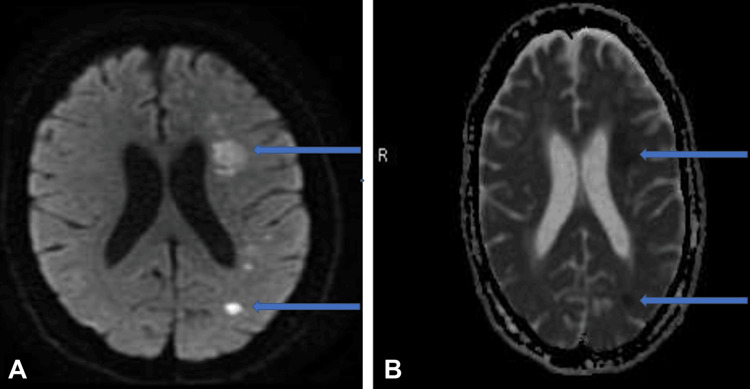
MRI brain (A) DWI and (B) ADC sequences showing multiple left hemispheric watershed infarcts (arrows) DWI: diffusion-weighted imaging; ADC: apparent diffusion coefficient

Blood glucose was maintained between 8 and 10 mmol/L using sliding-scale insulin; blood pressure was kept below 140/90 mmHg. The patient had a brain CT, which showed multiple recent/subacute lacunar infarcts in the left frontal white matter at the level of the corona radiata and centrum semiovale, left internal capsule, and anterior limb, in the background of mild generalized brain atrophy and small vessel disease, suspicious of tiny lacunar infarcts involving the right hemi-pons and marked soft tissue edema/thickening involving the left posterior upper cervical superficial muscle and the left sternocleidomastoid with air bubbles and overlying subcutaneous edema (Figure 1).

**Figure 2:**
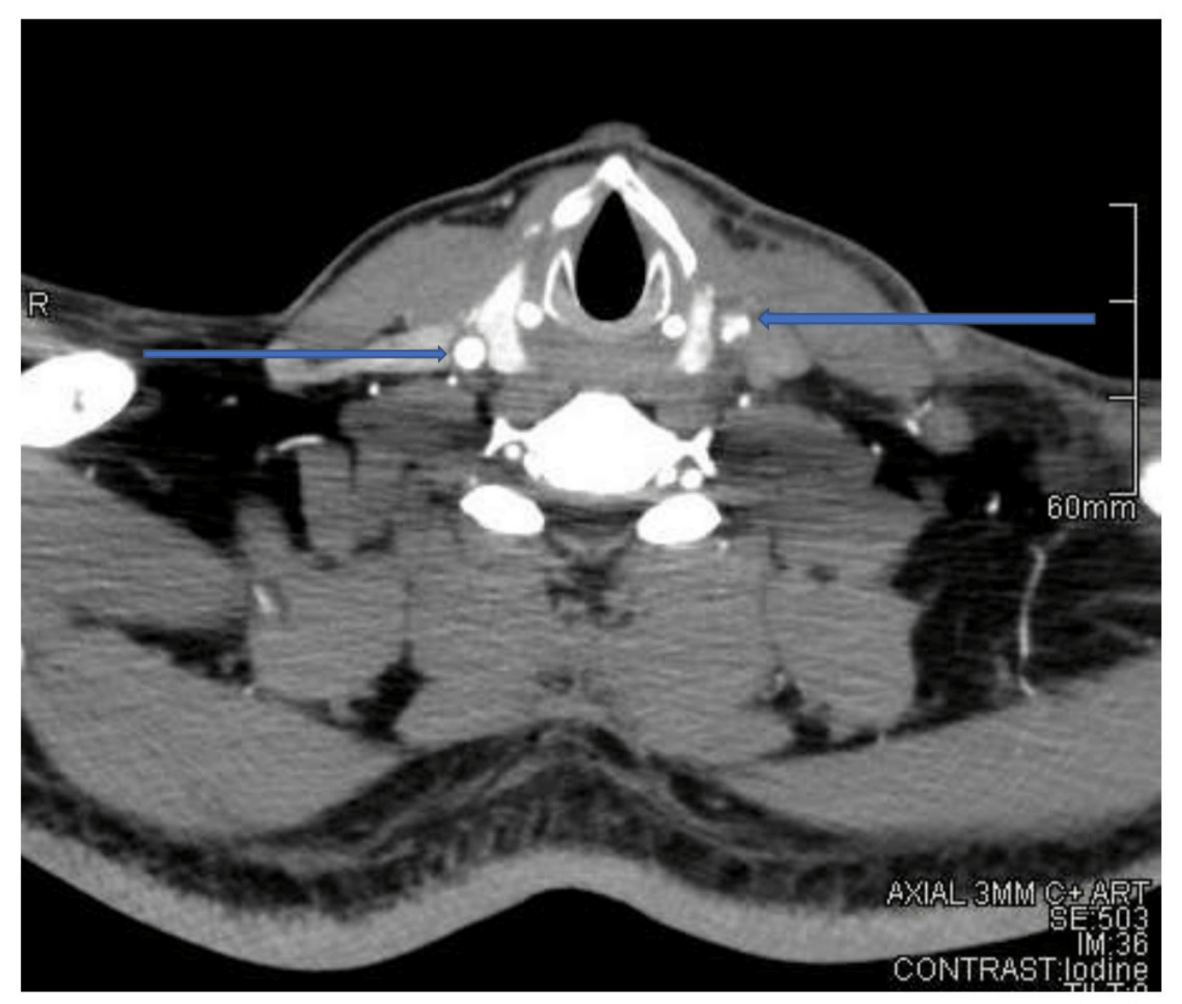
CT carotid angiogram (selected axial cut) showing abnormal left common carotid contour with associated luminal narrowing in comparison to the right

**Figure 3:**
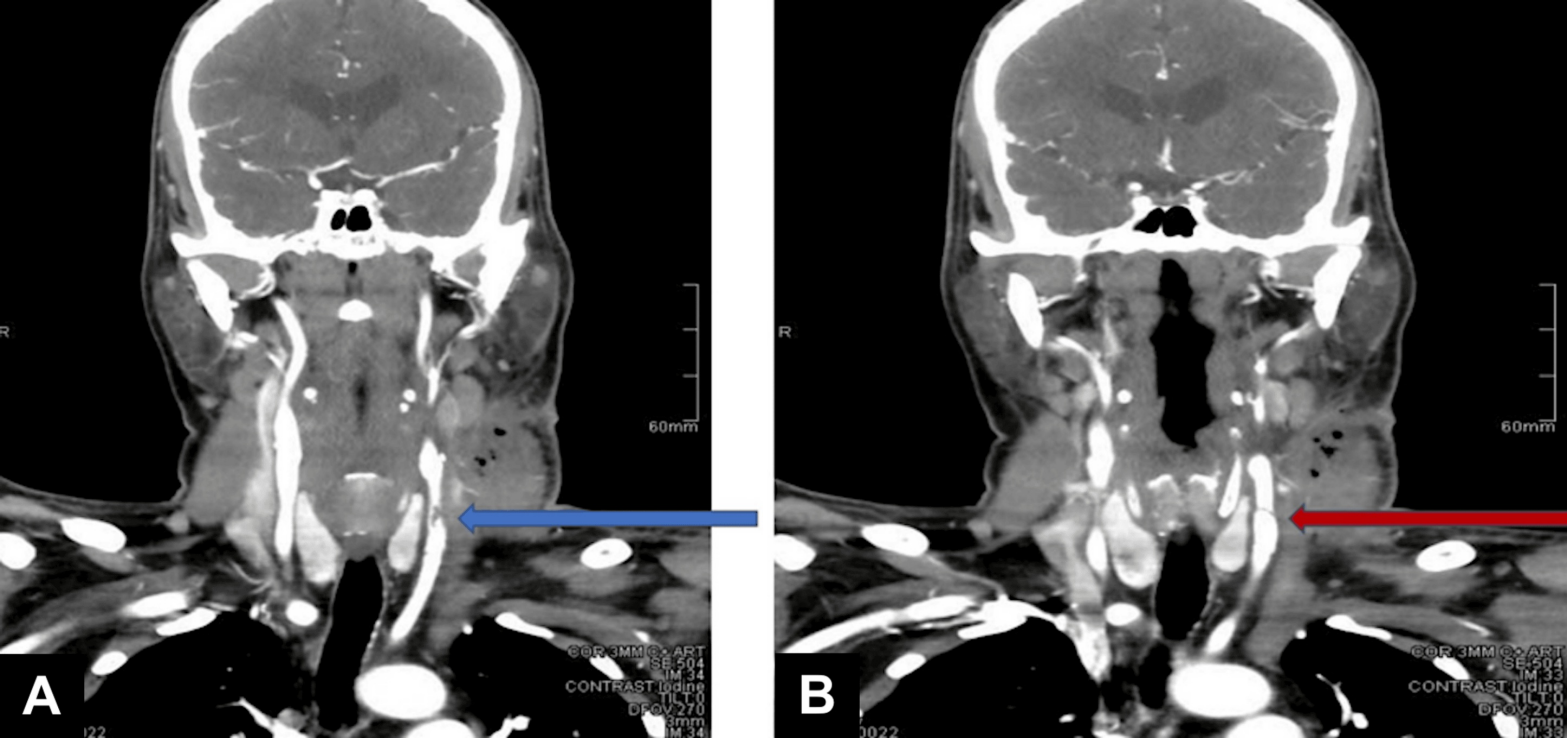
Selected coronal images of carotid angiogram showing (A) abnormal left CCA contour and luminal narrowing (blue arrow) with (B) associated intimal flab (red arrow) and patent internal carotid artery CCA: common carotid artery

**Figure 4:**
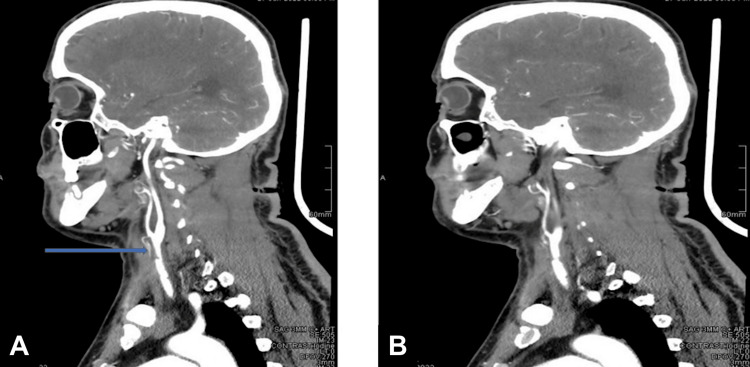
Selected sagittal cuts of carotid angiogram showing (A) left common carotid luminal narrowing and mural-based thrombus with (B) preserved distal ICA flow beyond the occluded common carotid segment ICA: internal carotid artery

**Figure 5:**
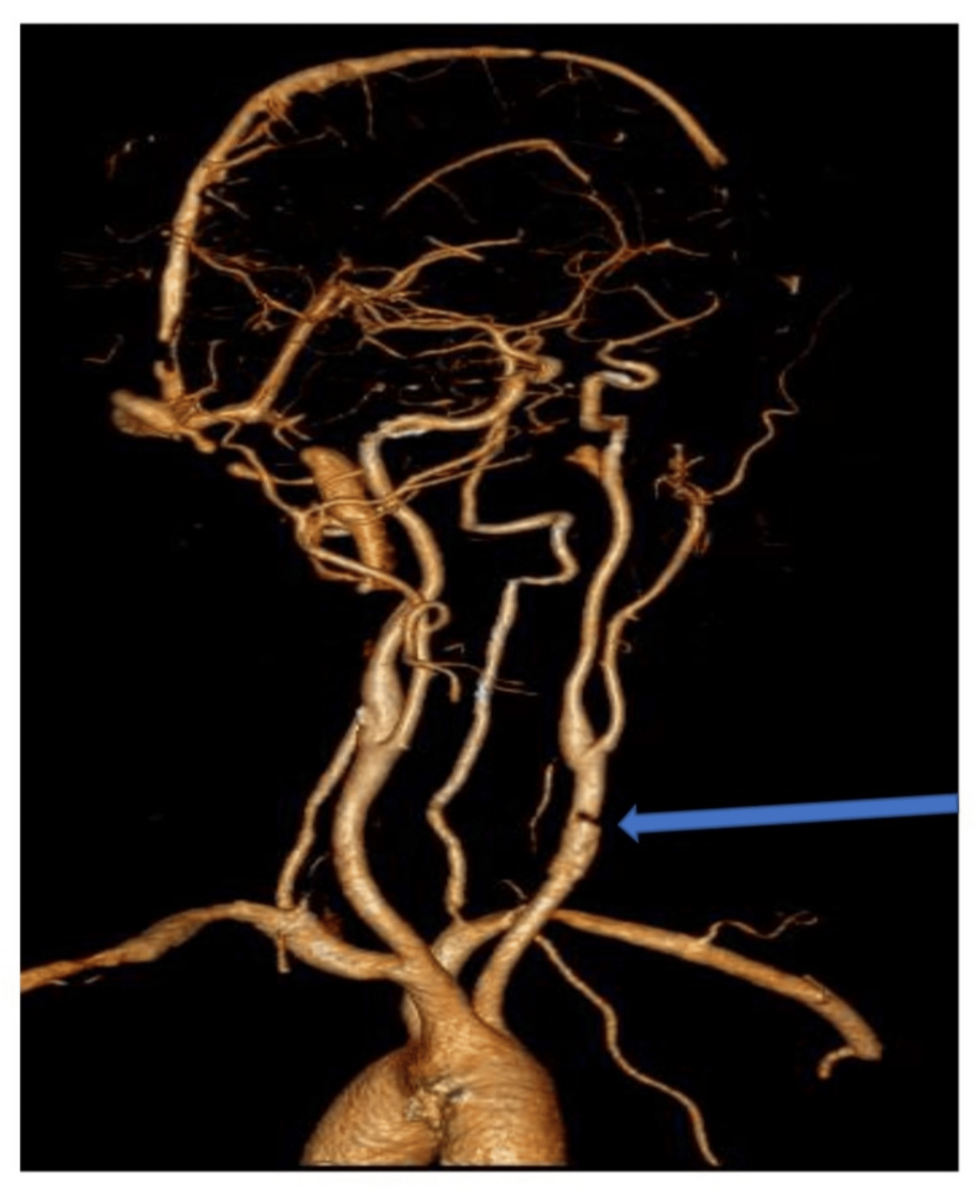
Carotid angiogram 3D reconstruction: focal discontinuity at the left common carotid artery with patent distal ICA ICA: internal carotid artery

A CT angiogram of the neck showed occlusion of the left common carotid artery from its origin to the bifurcation, with collateral flow through the external carotid artery. The basilar and vertebral arteries were normal (Figures 2-5).

Vascular surgery was involved, and surgical intervention was considered too high-risk due to the potential for hemorrhagic transformation of a stroke. A decision was made to prioritize conservative management. The patient was commenced on a therapeutic dose of low-molecular-weight heparin (enoxaparin 1 mg/kg subcutaneously every 12 hours) with close monitoring of the coagulation profile. The patient received IV piperacillin/tazobactam 4.5 g IV every eight hours for four days and IV vancomycin 1 g IV every 12 hours for five days. Following clinical improvement, the patient gradually improved with a GCS of 15/15 and had a mild residual hemiparesis. He was discharged on apixaban 5 mg twice per day. It is typically recommended that the duration of treatment be three to six months, depending on vessel recanalization and clinical progress. Follow-up vascular imaging was planned to determine the optimal duration of anticoagulation and to assess the efficacy of ciprofloxacin and Augmentin for a 10-day course. The patient was referred to physiotherapy and was followed up with at the outpatient clinic.

## Discussion

Head injuries due to camel bites are unusual, and such cases have mostly been reported in the Middle East. Similar incidents have been documented in Rajasthan, a desert region in India [3]. As demonstrated in this instance, these injuries can result in symptoms that range from minor infections to serious vascular and neurological complications. Since such scenarios are uncommon, it is essential to understand the possible outcomes of camel bites to anticipate potential challenges. It has been proposed that a camel bite could cause a stroke through either direct brain injury from the camel's teeth or complications like thrombosis, vasospasm, or hemorrhage.

In addition to the infections and neurological involvement, camel bites to the head and neck are dangerous and complicated because of the high vascularity in these areas, which makes surgical intervention difficult [4]. A study on vascular damage from camel bites was conducted in India. Out of the 23 arterial injury cases that were documented, only two right-sided neck bite cases involving occlusion of the right internal carotid artery with a hemispherical infarct were described; the remaining neck bite injuries did not involve any major vessels [5]. In our case, the internal jugular vein thrombosis after a bite and the left common carotid artery occlusion highlight the value of early imaging and multidisciplinary care involving medical, surgical, and radiological teams. The neurologic complications that our patient experienced sit with the limited literature on camel bite injuries. However, the varying appearances and outcomes of reported cases highlight the necessity of individualized patient care. The imaging in our case confirmed the connection between the bite and neurovascular events.

On the other hand, infections can arise from camel bites and can be polymicrobial, like *Staphylococcus* species, *Aeromonas*, *Pasteurella aerogenes*, and *Actinobacillus* [6]. Therefore, broad-spectrum antibiotic coverage is essential, along with tetanus and rabies vaccination. Public awareness campaigns are necessary as part of preventative measures for camel-related accidents, especially in regions where camels are prevalent. The risk of camel bites and the injuries they cause can be significantly reduced by following proper instructions to maintain a safe distance from domestic camels and by wearing appropriate safety equipment.

## Conclusions

Although camel bites are rare, they can be fatal. Camel bites to the neck with internal jugular thrombosis and common carotid artery occlusion are rarely reported. This case report demonstrates the serious vascular and neurological complications that can arise from camel bite injuries. The complexity of this case, which necessitates wound care, infection control, neurological management, and vascular thrombosis therapy, emphasizes the importance of early diagnosis, imaging, and multidisciplinary care. More studies and collaboration are required to better understand the pathophysiology of strokes caused by camel bites and to develop prevention and treatment options.
